# Minocycline-Induced Gum Pigmentation during Treatment for Acne Vulgaris

**DOI:** 10.1155/2022/9493061

**Published:** 2022-10-14

**Authors:** Jami Wang, Isabella Brown, Heidi Goodarzi

**Affiliations:** ^1^Western University of Health Sciences, Pomona, USA; ^2^Brigham Young University-Hawaii, Laie, USA; ^3^Hoag Hospital, Orange County, Newport Beach, USA

## Abstract

Minocycline, a type of tetracycline, is a broad-spectrum antibiotic that is commonly prescribed in dermatology for the treatment of acne vulgaris. Common side effects of minocycline include nausea, vertigo, and dizziness while less common side effects include hyperpigmentation. In this case study, we found an 18-year-old female who presented with dark blue pigmentation in her upper gum after using minocycline on and off for 4 years. After discontinuation of the minocycline for 2 years, the pigmentation decreased gradually.

## 1. Introduction

Minocycline is a broad-spectrum oral antibiotic, classified as a semisynthetic tetracycline, that has been commonly used in the treatment of acne vulgaris and sexually transmitted diseases [1]. While minocycline is known to be safe with little side effects, the most common ones include nausea, vertigo, and dizziness. A rare, yet severe, documented side effect of minocycline includes intracranial hypertension within 8 weeks of starting the treatment [2–4]. Minocycline can also cause skin hyperpigmentation under prolonged use of several months of treatment or at high doses [1, 5]. Hyperpigmentation can be found in any organ system, including the skin, lips, teeth, oral mucosa and gingiva, conjunctiva, and sclera [6]. The most common site includes the bones of the oral cavity, which affects more than 10% after 1 year and 20% of after 4 years of minocycline therapy [7, 8]. Pigmentation of oral soft tissue, including gingiva, is typically blue-gray or brown in appearance and less commonly documented [9]. The oral cavity is commonly overlooked and infrequently monitored for adverse effects, as minocycline is typically prescribed by physicians as opposed to dentists [10].

The 4 types of clinical presentations include: (i) dark blue-black macules near the site of inflammation, (ii) blue-gray pigments on the forearms and shins, (iii) muddy brown discoloration in sun exposed area known as “muddy skin syndrome” or “dirty skin syndrome,” (iv) muddy brown discoloration on pre-existing scars that are not sun exposed [11, 12]. Discontinuation of the therapy is recommended following minocycline-induced skin pigmentation.

### 1.1. Mechanism

The mechanism and pathophysiology of minocycline pigmentation is unknown. Minocycline is an unique tetracycline, as it has up to six metabolites with a variety of antibacterial properties [13]. The main metabolites include 9-hydroxyminocycline and two types of mono-N-demethylated derivatives [14]. There are several proposed mechanisms regarding hyperpigmentation caused by minocycline. One proposed mechanism of the hyperpigmentation is due to the accumulation of the minocycline derivatives that chelate to iron, calcium, and melanin [15, 16]. Another proposed mechanism includes the formation of insoluble salts from minocycline, which is accumulated in different locations [17].

### 1.2. Published Cases

Although it is an uncommon finding, there has been previously published case studies of minocycline-induced pigmentation of the gingiva. LaPorta et al. described a case of brown pigmentation of the upper right facial gingiva then spread to the lips and nail beds after 6 months of minocycline therapy. A biopsy showed that the gingiva and lip had increased melanin and melanocytes in both the epithelium and connective tissue [9]. In another case study, Tosios et al. presented a patient with pale brown pigmentation with a dark spot on the gingiva around the lower right central incisor after 5 months of minocycline treatment. The pigmentation also spread to the maxilla, mandible, and left lateral incisor and canine. A biopsy showed an increased amount of melanin in the basal and parabasal layers [18]. Furthermore, Meyerson et al. reported two women with isolated lingual hyperpigmentation caused by minocycline therapy. Both women had acquired pigmentation on their tongues after the use of minocycline for acne and rosacea [19].

While minocycline has been found to be associated with various adverse effects, the only adverse effect correlated with dosage is pigementation.. Goulden et al. evaluated the long-term side effects of minocycline for 700 patients with acne vulgaris, with dosing ranging from 100 mg to 200 mg daily. All patients in the study who developed pigmentation had a cumulative dose of over 70 g [16]. In addition to the high cumulative dose, the time frame for pigmentation to develop usually takes several weeks to years.

## 2. Case Report

An 18-year-old female patient presented to a dermatology clinic with an adverse effect of gingival hyperpigmentation caused by minocycline. The patient initially presented with a chief complaint of moderate comedonal and inflammatory hormonal acne on her face for several years. The patient reported no pertinent medical or surgical history, other than surgical wisdom teeth removal two years prior. The patient denied use of alcohol and tobacco. She had no known drug allergies. For the acne treatment, the patient had taken minocycline for an undisclosed amount of time, starting at 100 mg daily and increasing to 200 mg daily. The patient found minocycline to be effective for acne and wanted to continue. She was noted to have elements of hormonal acne so, after discussion with the patient, the treatment plan was to taper her off minocycline and start her on a low dose of spironolactone. The dosage of minocycline was decreased to 55 mg extended-release per day and spironolactone 100 mg oral tablet daily was started. The patient was recommended to follow-up in a month.

**Figure 1:**
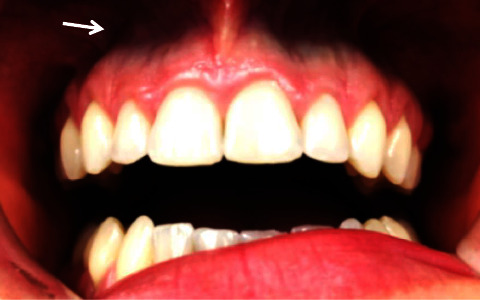
Dark blue pigmentation is found in the maxillary gingival margins.

One month later during the follow-up visit, the patient's medical records were obtained which documented that she was on and off minocycline for 4 years. Upon a full clinical examination, the patient's maxillary gingival margins noted to have dark blue pigmentations (Figure 1) but with normal texture and without edema. It was suspected that the dark blue pigmentation of the gums was likely a side effect of minocycline medication for a long duration of treatment. We did not note additional pigmentation at other sites, including the skin, nails, and ears.

When the patient started taking minocycline, the prescribed dosage was taken every night. There were no additional side effects for completion. The updated treatment plan was to discontinue minocycline immediately and increase the spironolactone. The expected outcome for discontinuing minocycline would be a reduction of pigmentation over the next few months to years. In the second follow-up visit, the patient continued with spironolactone and topical treatments.

**Figure 2:**
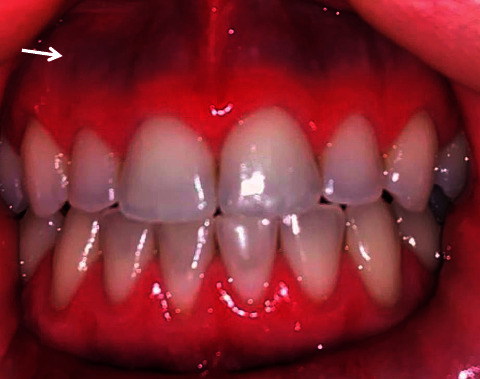
1.5-year follow-up after discontinuation of minocycline, dark blue pigmentation is found in the maxillary gingival margins.

**Figure 3:**
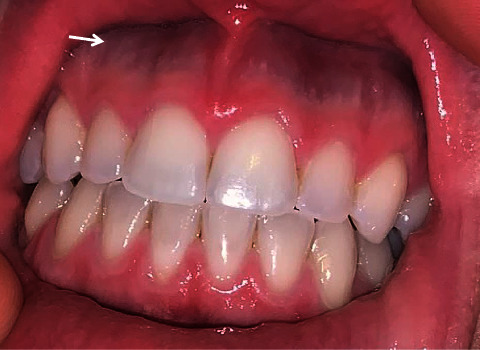
2-year follow-up after discontinuation of minocycline, dark blue pigmentation is found in the maxillary gingival margins.

In the 1.5-year follow-up after discontinuation of the minocycline treatment, the patient still had dark blue pigmentation in the gums (Figure 2), although lighter in pigmentation. The patient was lost to follow up but later informed us that she discontinued spironolactone, started and discontinued oral hormonal contraceptives, tea tree, calendula, yarrow, and parsley extracts. She opted for lifestyle changes including liothyronine for weight loss. In the 2-year follow-up call after discontinuing minocycline treatment, the dark blue pigmentation in the gums remained (Figure 3). There is no noticeable change in the pigmentation from the 1.5-year follow-up. Given the patient had no additional clinical symptoms, a biopsy would be inappropriate at this time. Diet and lifestyle changes are still consistent.

## 3. Discussion

In dermatology, minocycline is an antibiotic that is commonly prescribed to treat acne vulgaris that does not respond to topical treatment. Recent advancement in using minocycline for moderate to severe acne vulgaris has been shown to be effective and safe in a topical foam form. However, the side effect of skin discoloration was comparable for both a tablet and topical treatment [20]. The main treatment for minocycline-induced pigmentation is the cessation of therapy, which would allow the pigmentation to resolve over the course of several months to years [21, 22]. Aside from discontinuing the medication, additional treatments are limited. A few case studies have shown that selective photothermolysis therapy with a variety of Q-switched lasers can be helpful in reducing the hyperpigmentation as well [23–25].

Minocycline is reported to have a higher frequency of skin pigmentation than other tetracyclines, such as doxycycline. One potential reason is that minocycline is more commonly used in long-term treatments compared to other tetracyclines that are more often used for acute conditions [21, 26]. This is because the American Academy of Dermatology recommends minocycline, and doxycycline, as first-line oral antibiotics therapies in the treatment of severe acne vulgaris [5, 27, 28].

There are limited studies on the association between the length of minocycline use and the development of hyperpigmentation. However, in one systematic review, Bienefeld et al. showed the treatment of antibiotic therapy should not exceed 12 weeks due to antibiotic resistance [29]. In another retrospective study, Fay et al. 44 of the 121 (36%) rheumatoid arthritis patients receiving more than 1 month treatment of minocycline developed skin pigmentation [30].

## 4. Conclusion

Minocycline-induced pigmentation is a side effect that dermatologists should be aware of when treating for acne vulgaris. Although the pigmentation can likely be reversed after discontinuation of minocycline, it is important for the patients to be educated prior and monitored throughout their treatment. A certain amount of time should be allotted to taking the drug under the same physician for continued monitoring of the side effects. While the pigmentation does not have any life-threatening side effects, the pigmentation can still affect the patients' daily life. In our case study, the patient explained that the minocycline-induced pigmentation caused a “pull back in self-confidence” as the patient became “afraid to smile too big.” It is important to understand these side effects can have lasting effects for patients beyond their treatment time. There have been several other case studies that have examined gingival pigmentation touch on how through a smile, one can convey happiness, satisfaction, achievement, self-confidence and more [31]. The minocycline-induced pigmentation can also be mistaken as clinical manifestations of other diseases the patient does not have. Other medication-induced pigmentation include the use of tranquilizers, phenolphthalein, antimalarial medications, estrogen, chemotherapeutic agents, and medications used in the treatment of AIDS.

As a patient continues the use of minocycline, side effects and maintenance checks should be reviewed and maintained both directly to the patient and documented on their charts. While the exact mechanism of location for gingival discoloration is unclear, further analysis on the patients smile and sunlight exposure could be examined. A more severe complication to consider would be drug reactions with eosinophilia and systemic symptoms, also known as DRESS syndrome. DRESS syndrome is a condition in which there is a hypersensitivity reaction to a therapeutic medication. While the cause and clinical presentation of DRESS syndrome is largely variable, the most common symptoms include rash, fever, hematologic abnormalities, and involvement from internal organs. In most cases, the effects are seen within 1–8 weeks after the exposure to the suspected medication [32].

Minocycline-induced pigmentation should continue to be studied and analyzed to understand the best method of preventing discoloration. Healthcare providers should inform patients of unwanted side effects prior to starting the medication.

## Data Availability

The clinical notes data used to support the findings of this study are available from the corresponding author upon request.
